# The Year Book of Treatment, 1894

**Published:** 1894-05

**Authors:** 


THE YEAR BOOK OF TREATMENT, 1894. Lea Brothers & Co., Philadelphia.

This, the tenth of the series, contains two new articles, one "Medical Diseases of Children," by Dawson Williams; the other an article upon Bacteriology by William Hunter. The remainder of the book consists of extracts, chiefly from current English literature, and covers briefly a large field in which the reader will be likely to find a good many things which have escaped his notice in the medical journals. The summary of the therapeutics for 1892 and 1893 is to be commended as taking up but a few of the many hundreds of new remedies, so-called, with which the profession has been annoyed. L. H. J.

